# Whole-Genome Sequence of a Colombian *Acinetobacter baumannii* Strain, a Coproducer of OXA-72 and OXA-255-Like Carbapenemases

**DOI:** 10.1128/genomeA.01558-16

**Published:** 2017-02-16

**Authors:** Sandra Yamile Saavedra, Diego Prada-Cardozo, Verónica Rincón, Hermes Pérez-Cardona, Andrea Melissa Hidalgo, María Nilse González, María T. Reguero, Emilia M. Valenzuela de Silva, José R. Mantilla, Laurent Falquet, Emiliano Barreto-Hernández, Carolina Duarte

**Affiliations:** a Microbiology Group, National Institute of Health, Bogotá, Colombia; b Bioinformatics Group, Biotechnology Institute, Universidad Nacional de Colombia, Bogotá, Colombia; c Molecular Epidemiology Laboratory, Biotechnology Institute, Universidad Nacional de Colombia, Bogotá, Colombia; d Biochemistry/Bioinformatics Unit, Université de Fribourg, Fribourg-Suiza, Switzerland

## Abstract

Colombian *Acinetobacter baumannii* strain ST920 was isolated from the sputum of a 68-year-old male patient. This isolate possessed *bla*OXA-72 and *bla*OXA-255-like genes. The assembled genome contained 4,104,098 pb and 38.79% G+C content. This is the first case reported of the coproduction (*bla*OXA-72 and *bla*OXA-255-like) of carbapenem-hydrolyzing class D β-lactamases (CHDLs) in *Acinetobacter baumannii*.

## GENOME ANNOUNCEMENT

The resistance to carbapenemases in *Acinetobacter baumannii* has primarily been associated with the production of carbapenem-hydrolyzing class D β-lactamases (CHDLs). Six different groups of CHDLs have been described for *Acinetobacter baumannii*, where the OXA-51-like group is intrinsically chromosomal, while the other groups are acquired: OXA-23-like, OXA-40/24-like, OXA-58-like, OXA-143-like, and OXA-235-like (1, 2). In Latin American, the high prevalence of multidrug resistant *A. baumannii* strains is one of the principal causes of health care-associated infections (HAI) (3–7).

As part of the HAI surveillance program, the Microbiology Group of the National Institute of Health of Colombia isolated the GMR_RB_1399 strain in May 2014 from the sputum of a 68-year-old male patient. This strain was identified as *Acinetobacter baumannii* using the automatic system Phoenix (Becton Dinckinson) and its antimicrobial susceptibility testing (*E* test, bioMérieux) showed resistance to imipenem, meropenem, doripenem, and piperacillin-tazobactam and susceptibility to ceftazidime, cefepime, ampicilin-sulbactam, ciprofloxacin, amikacin, gentamicin, and colistin according to the Clinical and Laboratory Standards Institute (8). Therefore, this strain was classified as multidrug resistant (MDR) according to the standardized international terminology (9).

The genomic DNA was extracted using the QIAamp DNA minikit (QIAGEN). The DNA quantification was performed using Quant-iT PicoGreen dsDNAPicogreen (Invitrogen), using Victor three fluorometry (PerkinElmer). The library was constructed with the TruSeq DNA PCR-free sample preparation kit (Illumina). This library was sequenced using the Hiseq2000 system (Illumina).

A total of 4,395,897 paired-end reads were obtained, with an average length of 101 pb. *De novo* assembly was carried out using SPAdes version 3.8 (10), resulting in 24 contigs, 4,104,098 pb, 180× coverage and 38.79% G+C content.

The GMR_RB_1399 strain was identified as a new sequence type (ST) by multilocus sequence typing (MLST) analysis using the Pasteur scheme (CGE server). The strain was registered in the pubMLST database (http://pubmlst.org/abaumannii/submission.shtml) as an ST920.

The annotation process was performed using Prokka software (11), which was enriched with the following databases: Resfam (12), CARD (13), Gibsy (14), and VFDB (15). Prokka annotated 3,785 coding sequences (CDSs), three rRNAs, 65 tRNAs, one transfer-messenger RNA (tmRNA), and 3,854 genes.

The genome annotation showed the following carbapenemases: *bla*OXA-72 (OXA-40/24-like), *bla*OXA-255-like (OXA-143-like), and *bla*OXA-106-like (OXA-51-like). *bla*OXA-106-like presented a single amino acid change (Thr97Ser).

In different studies, OXA-72-producing *Acinetobacter baumannii* isolates showed resistance to all β-lactams, included carbapenemes (16–22). It was first reported in Thailand (23) but has now spread to several continents (16–27), including South America (22, 23, 26, 27) and Colombia, where there is already a report of its presence in an *Acinetobacter baumannii* strain (22).

This is the first case reported in Colombia of the presence of OXA-255-like (OXA-143-like) and the first report of its presence in *Acinetobacter baumannii* ever. *bla*OXA-255-like had two amino acid changes (Ser158Asn and Ala183Val), differs from OXA-143 in 18 amino acids and was found previously in *Acinetobacter pittii* with a carbapenem-resistant profile (27–29).

Additionally, this is also the first case reported of the coproduction (*bla*OXA-72 and *bla*OXA-255-like) of CHDLs in *Acinetobacter baumannii*.

### Accession number(s).

This whole-genome shotgun project has been deposited at DDBJ/EMBL/GenBank under the accession no. MPPK00000000.
